# Incorporating FRAX into a nurse-delivered integrated care review: a multi-method qualitative study

**DOI:** 10.3399/BJGPO.2022.0146

**Published:** 2023-03-22

**Authors:** Ashley Hawarden, Laurna Bullock, Carolyn A Chew-Graham, Daniel Herron, Samantha Hider, Clare Jinks, Risni Erandie Ediriweera De Silva, Annabelle Machin, Zoe Paskins

**Affiliations:** 1 School of Medicine, Keele University, Keele, UK; 2 Department of Psychology, School of Health, Science and Wellbeing, Staffordshire University, Staffordshire, UK; 3 Haywood Academic Rheumatology Centre, Midlands Partnership NHS Foundation Trust, Staffordshire, UK; 4 Department of Family Medicine, Faculty of Medicine, University of Colombo, Colombo, Sri Lanka

**Keywords:** osteoporosis, multimorbidity, primary health care, review, fracture, qualitative methods

## Abstract

**Background:**

People with inflammatory rheumatological conditions (IRCs) are at increased risk of common comorbidities including osteoporosis.

**Aim:**

To explore the barriers to and facilitators of implementing nurse-delivered fracture risk assessments in primary care, in the context of multimorbidity reviews for people with IRCs.

**Design & setting:**

A multi-method qualitative study in primary care.

**Method:**

As part of a process evaluation in a pilot trial, semi-structured interviews were conducted with 20 patients, two nurses, and three GPs. Twenty-four patient–nurse INCLUDE review consultations were audiorecorded and transcribed. A framework analysis was conducted using the Theoretical Domains Framework (TDF).

**Results:**

Nurses reported positive views about the value of the Fracture Risk Assessment Tool (FRAX) and they felt confident to deliver the assessments following training. Barriers to implementation, as identified by TDF, particularly related to the domains of knowledge, skills, professional roles, and environmental context. GPs reported difficulty keeping up to date with osteoporosis guidelines and voiced differing opinions about whether fracture risk assessment was the role of primary or secondary care. Lack of integration of FRAX into IT systems was a barrier to use. GPs and nurses had differing views about the nurse role in communicating risk and acting on FRAX findings; for example, explanations of the FRAX result and action needed were limited. Patients reported limited understanding of FRAX outcomes.

**Conclusion:**

The findings suggest that, with appropriate training including risk communication, practice nurses are likely to be confident to play a key role in conducting fracture risk assessments, but further work is needed to address the barriers identified.

## How this fits in

Primary care plays a central role in identifying and assessing people with or at risk of osteoporosis and in providing long-term management. This qualitative study identified that implementing FRAX in primary care integrated reviews is possible but more support is needed with clinical decision making, risk communication, clarifying professional roles, and IT integration.

## Introduction

Osteoporosis is characterised by low bone mass and disruption of bone microarchitecture leading to bone fragility and fractures.^1^ Fragility fractures (fractures resulting from low trauma) may result in pain, disability, and reduced quality of life. People with osteoporosis may be managed with a combination of lifestyle modification, falls prevention interventions, and osteoporosis medication. Evidence-based osteoporosis medications to reduce fracture risk are recommended by the National Institute for Health and Care Excellence (NICE), which are readily available, cost and clinically effective, and reduce fracture risk by 20%–70% depending on fracture site.^2^

The first step to manage people with osteoporosis is to identify who is at risk. Fracture risk assessment has been shown to be cost and clinically effective in UK primary care;^3^ however, in the UK specifically, it is estimated that 65% of females seen in primary care and at high risk of fragility fracture do not receive appropriate management, which is suggested to be as a result of deficits in identification, diagnosis, and awareness.^4^ There is, therefore, an urgent need to address barriers to and facilitators of fracture risk assessments in primary care.

People with IRCs, such as rheumatoid and psoriatic arthritis, are known to be at an increased risk of osteoporosis and fragility fracture.^5,6^ National guidelines suggest patients with rheumatoid arthritis should have an annual review, which includes screening for comorbidities such as cardiovascular disease, osteoporosis, and depression.^7^ Annual reviews are not part of standard care for other IRCs despite similar increased risks of comorbidities. In the UK, although most patients with IRCs will see a rheumatologist in secondary care for the management of their condition, arguably GPs in primary care have more experience in assessment and management of comorbidities such as cardiovascular disease.

The INtegrating and improving Care for patients with infLammatory rheUmatological DisordErs (INCLUDE) review pilot trial therefore aimed to evaluate the feasibility and acceptability of a nurse-delivered review in primary care for people with IRCs to identify and manage common comorbidities.^8^ This holistic review included case-finding, identification, and initial management of fracture risk, mood disorders, and cardiovascular risk factors. The results from the pilot study reporting quantitative outcomes and addressing feasibility and acceptability of the review are already published.^9,10^ However, this study provided a unique opportunity to also specifically explore the barriers to and facilitators of implementing the fracture risk assessment component of the integrated review from the perspective of patients and clinicians, which was the aim for this study.

## Method

This study utilised data from patient and clinician semi-structured interviews and recorded consultations from the INCLUDE study.^8^

The content of the INCLUDE review was developed with patients and practitioners.^8^ INCLUDE study nurses underwent training to include case-finding, and identification and assessment of cardiovascular disease risk, anxiety and depression, and fracture risk, including the use of simulated patients. The fracture risk component of the review utilised FRAX;^11^ a computer-generated algorithm showing the 10-year probability of hip and major osteoporotic fracture, aiding clinicians and patients in decision making regarding osteoporosis investigation and management.^2^

### Sampling and recruitment

Patients with IRCs were invited to attend an INCLUDE review. With appropriate consent, a random sample of patient–nurse INCLUDE consultations were audiorecorded. Fidelity checks were undertaken of the recorded consultations to demonstrate which parts of the INCLUDE review were delivered using a bespoke checklist.^10^ Following the INCLUDE review, patients (*n* = 55) were purposively invited via post to take part in a semi-structured interview to explore their experiences of the INCLUDE review.^9^ The nurses delivering the INCLUDE review (*n* = 2) were invited to take part in two semi-structured interviews; one after training, but before completing any INCLUDE consultations, and the other towards the end of study. GPs within participating practices were also invited to take part in a semi-structured interview.

### Procedures and data collection

Interviews were conducted by an experienced qualitative researcher. Topic guides (see Supplementary Appendices S1–S4), designed by the study team and informed by public contributors, facilitated exploration of participants’ perceptions of the acceptability of the INCLUDE review.^8,10^ Interviews, which lasted between 30 and 121 minutes, were audiorecorded, transcribed verbatim, and anonymised. Consultation recordings relating to fracture risk and bone health were anonymously transcribed verbatim.

### Data analysis

Data were extracted from anonymised interview and consultation transcripts relating to osteoporosis and/or fracture risk. Extracted data were analysed using a systematic framework approach using the TDF. The TDF is a framework for understanding behaviour change in the implementation of new interventions and consists of 14 domains, including the following: knowledge; skills; professional role and identity; beliefs about capabilities; optimism; beliefs about consequences; reinforcement; intentions; goals; memory, attention and decision processes; environmental context and resources; social influences; emotion; and behavioural regulation.^12,13^ Interview and consultation transcript extracts were checked against the audiorecordings before being read and re-read to facilitate data familiarisation. Data were deductively mapped using a framework matrix to the TDF by two authors (a rheumatologist and experienced qualitative researcher, and an experienced qualitative researcher) to facilitate understanding of barriers to and facilitators of an osteoporosis review in primary care. Findings were discussed as a multidisciplinary team to contribute to interpretation.

## Results

In total, 24 patient–nurse INCLUDE review consultations were audiorecorded. Twenty patients (16 females and four males) with a mean age of 68 years (range 35–80 years) were interviewed, and 19 of the interview transcripts contained reference to osteoporosis and were included in this analysis (Table 1). Three GPs in participating practices and the two nurses delivering the INCLUDE review took part in semi-structured interviews. Given the small sample size, GP and nurse demographics are not reported to preserve anonymity.

**Table 1.** Patient participant (interviewee) demographics

Patient participant characteristics	*n* ^a^
Age, years	
30–59	5
60–89	15
IRC^b^	
Rheumatoid arthritis	6
Psoriatic arthritis	4
Ankylosing spondylitis	4
Polymyalgia rheumatica	5
Giant cell arteritis	2
Sex	
Male	4
Female	16

^a^One patient included in this table was not included in the analysis as their interview transcript did not make reference to osteoporosis or fracture risk.^b^One patient reported two IRCs. IRC = inflammatory rheumatological condition.

Illustrative quotations are presented below, according to the relevant TDF domains from consultation recordings (CR), patient (P), nurse (N), and GP interviews. Nurse quotations also specify the interview timing; interview completed after the INCLUDE training but before starting INCLUDE reviews (PRE) or after completing INCLUDE reviews (POST). At times, TDF domains have been presented together if findings across domains were interrelated. The participant identifier codes and completed TDF analysis matrix can be seen in Supplementary Box S1.

### Knowledge

During the consultation, some patient participants told the nurse that they had limited prior knowledge of osteoporosis. Following the INCLUDE review patient participants reflected that the review had increased their knowledge and understanding of osteoporosis, with nurses also suggesting that patients had a greater understanding of osteoporosis following the review:

*'I’ve probably become more aware of that. I am aware of* [osteoporosis] *now, from the review*.*'* (P494)

Other patient participants were unable to recall discussions regarding osteoporosis or the calculation of fracture risk, or, had uncertainty regarding what osteoporosis was and sometimes confused it with osteoarthritis:

*'They obviously said its thinning of your bones. It can cause osteoarthritis; I can’t remember what else they said*.*'* (P10)

In the interviews, patients expressed a desire for more information regarding osteoporosis management, such as patient information leaflets covering drug treatments, diet, and exercise:

*'Some more information might be useful if somebody needed treating, not just me but other people, you know dietary information or osteoporosis.'* (P51)

Despite patient participants having risk factors that increase the risk of low-trauma fracture (for example, steroid use), some focused on osteoporosis being inevitable:

*' …* [the steroids] *hadn’t affected it, you know, or that much anyway* […] *If your bones get thin your bones get thin, don’t they? Some people are more susceptive to that sort of thing. I just hope I’m built like me mother.'* (P564)

GPs reflected on their perceived lack of knowledge related to osteoporosis management, which was attributed to limited experience and repeated changes in clinical guidelines:

*'The osteoporosis bit is a little bit hard for us because it keeps changing* […] *it’s just measuring the risk of osteoporosis and what to do next it’s a bit less clear.'* (GP05)

### Skills and beliefs about capabilities

The nurses’ skills communicating risk influenced their perceived capability to deliver the fracture risk assessment. Both nurses reported no prior experience using the FRAX tool, with both nurses reflecting that, before the INCLUDE training, they would not have been able to communicate fracture risk to patients:

*'I wasn’t quite sure how to explain to the patient about their risk. I already knew a lot about the advice to give. But when you’re doing it all the time it’s hard to think actually what you do say.'* (N02PRE)

When reflecting on the INCLUDE training, the nurses were positive about the inclusion of simulated patients. Nurses reported that the training increased their understanding of the FRAX template, their capability and confidence in its use, and that they had the required skills to communicate to patients their risk of future fracture.

During the consultation, nurses communicated the risk of future fracture outputted by FRAX as a percentage and by showing the results on the screen:

N: *'Let’s calculate. 5.8% risk. I will show you the screen*.*'* (CR520)

In interviews completed after delivering INCLUDE reviews with patients, nurses reflected on the difficulties they faced; for example, when interpreting some of the FRAX assessment questions, such as what constituted a previous fracture:

*'There’s a question on FRAX that I just overlook over time, there’s one strange question* […] *I’ve mentioned it to* [N02]*, who’s obviously my co-worker on this, and they’re like, "Yeah, I don’t – I’m not quite sure what that is."'* (N01POST)

Aside from FRAX, one nurse also reflected on their inability to access previous bone health investigations in the patient record, hindering their confidence when providing recommendations. This, however, was not a problem for one nurse completing the INCLUDE review, as illustrated by consultation recordings:

N: *'I noticed on your records that you’ve had a DXA* [dual-energy X-ray absorptiometry] *scan in the last couple of years. Is that right?* […] *Let me have a look on your records. I am sure I have seen that somewhere. Hip DXA 2015*.*'* (CR90)

One GP suggested that patients who consulted with them after the nurse INCLUDE review did not have a clear understanding of risk factors associated with osteoporosis or their risk of future fracture:

*'... they* [patients] *certainly didn’t come with a clear understanding of what the risks were and to what extent they were modifiable*.*'* (GP12)

When considering future implementation of the INCLUDE review, GPs questioned whether practice nurses had the skills or capability to deliver an osteoporosis risk assessment without training:

*'Well they* [untrained nurses] *couldn’t do the exact* [review] *that is being done here because they wouldn’t know how to do the osteoporosis score* […] *they would have to be up to date on osteoporosis, which they won’t normally know much about*.*'* (GP05)

### Professional role and identity

The limitation of roles was a key area of discussion, with nurses considering osteoporosis management as outside the scope of a nurse role:

*'The signposting for me was always, "Go and discuss this now with a — you know, with a true medical professional, go and see the GP and erm, let them weigh up the risks for you." All I’m doing crucially here is case-finding.'* (N01POST)

N: *'So,* [medication] *is probably recommended. I am not going to recommend that you take that. I am not here to prescribe that. What I would recommend is that you speak to your GP about that*.*'* (CR467)

Nurses and GPs reported that for patients with IRCs, often under specialist secondary care, osteoporosis risk management should be within the remit of secondary care. Uncertainty regarding the roles of primary and secondary care was perceived to contribute to aspects of care being missed:

*'The general practice probably think a lot’s been done in secondary care, secondary care think a lot of things are being done in primary care and the things that are missed are the things that we’ve covered.'* (N01POST)

*'What extent does primary care have a role with these patients and to what extent is it done in secondary care? I think there was a little bit of uncertainty on the part of the primary care nurses as to exactly what they should be doing with these reviews.'* (GP12)

### Goals and optimism

Nurses and GPs perceived that a primary goal of the INCLUDE review was to identify patients at risk of fracture and osteoporosis who may have otherwise been missed during routine care. GPs were also optimistic that FRAX assessment in patients with IRCs would result in long-term patient benefit by preventing future fractures and supporting ‘proactive management’ of osteoporosis:

*'It was about spotting those gaps in care, in primary care, for people with inflammatory arthritis.'* (N01POST)

In accordance with the goal described by clinicians, patients reflected that the INCLUDE review directly enabled further investigations and treatment:

*'So, it’s all been triggered really so I wouldn’t have gone for any of these appointments to know that’s something happening with it, probably till it’s too late, so that was good*.*'* (P143)

### Beliefs about consequences

Some patients were positive about having a fracture risk assessment and the further investigations proposed during the review. While other patients expressed concerns that the review would lead to additional hospital visits or medications:

*'I thought the way I feel at the moment, I don’t want to do that* [go for further investigations] […] *I suspect I will get sent for it eventually but I’ve escaped it at the moment. Rightly or wrongly, you know, just didn’t want to really start taking something else.'* (P494)

### Reinforcement

The nurses reported that the National Osteoporosis Guidelines Group intervention threshold graph produced by the FRAX tool, which is calculated based on the patient’s personalised information, was useful to reinforce their conclusions and support discussion of risk:

*'You could show the patient where the cross was and say that this is you based on your individual circumstances so whether or not they smoked, whether or not they drink, their age or what condition they’ve got, erm so I’d say, you know, patients like to know that they are on the green, actually, that was very reinforcing* […] *I had quite a few in the amber, you know patients were quite happy to go and have a conversation with their doctor about bone protection.'* (N02POST)

Nurses described both positive and negative feedback from GPs following patient referral, despite all patients being identified as high risk of osteoporosis during the INCLUDE review:

*'I did get feedback from a GP that I’d referred to them saying they needed a bisphosphonate and the GP said to me, "Yeah, you were right, send that round." So that was quite reassuring*.*'* (N02POST)

*‘"You didn’t need to send them to me"* [imitating GP response] *and I thought I don’t want to look a bit of an idiot with that but I don’t know what it can do about it really*.*'* (N01POST)

### Intentions

One patient participant described manipulating the responses entered into FRAX to achieve a more favourable (low-risk) outcome. The patient described that both themselves and the nurse made a conscious decision to exclude a previous low-trauma fracture from the risk calculation:

*'But if you put the break in, then I scored, obviously completely different so we decided to leave the break out because I, on my say so really because the way I’ve broken it*.*'* (P494)

### Memory, attention, and decision processes

Nurses demonstrated complex decision making, taking into consideration the output of FRAX and patient’s IRC, to decide if further bone health investigation and/or treatment was appropriate:

N: *'It is suggesting you are quite low risk there. I’ll show you that. The higher up your cross the more at risk you are of osteoporosis. In green it is saying just consider lifestyle advice and reassure you with that. But, the fact that you have got the giant cell arteritis is telling me otherwise. It is telling me to consider that. I advise you to book an appointment with your GP to consider a bisphosphonate prescription*.*'* (CR518)

Similarly, one GP described the complexity of the decision-making process when considering if patients should be referred for further bone health investigations:

*'Like this man, for the DXA, he’s 90 so whether we do DXA scans on 90, I don’t know, but they just, I don’t think he’s still on steroids, I don’t think he still needs one, so it’s just a little bit of a grey area.'* (GP05)

### Environmental context and resources

When considering the environmental context and resources, nurses discussed the use of GP IT systems. Although the nurses, at times, faced difficulties interpreting FRAX questions (see beliefs about capabilities), one nurse suggested that the practical aspects of using FRAX, such as accessing the tool and entering patient information, was acceptable:

*'I think the FRAXscore was really straightforward actually* […] *I think there were no problems with FRAX at all* […] *I think it was very user-friendly, very easy to use.'* (N02POST)

The other nurse, however, described challenges owing to a lack of FRAX integration into existing GP systems, making it at times difficult to navigate between the two when inputting data.

### Social influences and emotion

Patients, on the whole, were satisfied with the review and spoke positively about the outcomes. The INCLUDE review provided the opportunity for one patient to receive, what they perceived to be, a necessary investigation:

*'I was thinking, "Different friends of mine have — they’ve had these bone scans, you know, and I’ve got this complaint and I’ve never had one." So, I was pleased about that*.*'* (P500)

A further patient reflected on their positive experience of the review, suggesting potential benefits of the INCLUDE review to investigate bone health and risk of fractures for their friends who also have IRCs:

*'I’ve got friends who have got rheumatoid arthritis who aren’t as lucky as me and I think, "Gosh" you know if they were in this chair instead of me you know maybe this would help them so much.'* (P112)

When considering the emotional response of patients, one nurse reflected on the importance of the INCLUDE resources to support informative, rather than fearful, risk communication:

*'It’s just using the right terminology, cause, you don’t want to scare a patient by saying, oh you know, you’re really at risk of this but if you explain it to them using the sort of vocab that they’ve given you, and the script, with the grid, it comes across much more informative*.*'* (N02PRE)

## Discussion

### Summary

Primary care plays a central role in identifying and investigating people with or at risk of osteoporosis and providing long-term management. This article presents an exploration of the barriers to and facilitators of implementing an integrated fracture risk assessment using FRAX in primary care from the perspective of patients and involved practitioners in the context of multimorbidity reviews for people with IRCs. It was found that patients and nurses were positive about the goals of the review and potential benefits. However, key barriers to delivering FRAX assessments in primary care were identified relating to clinical decision making, risk communication, professional roles, and integration of FRAX with existing IT systems.

### Comparison with existing literature

Clinicians reported some difficulty in clinical decision making in osteoporosis. The limited available evidence regarding GPs’ attitudes to and experience of managing people with osteoporosis, suggests a need to further support GPs’ knowledge and decision making. In a survey of 2515 GPs in the UK, despite 93% of GPs identifying osteoporosis as a condition of considerable importance, 88% of GPs reported minimal education during undergraduate training, and 54.5% reported minimal education during post-graduate training.^14^ A questionnaire study of 112 GPs in the UK found that 68% of GP participants, if consulting with a 55-year-old with low-trauma fracture, would not assess for or initiate treatment for osteoporosis, suggesting either lack of awareness or knowledge.^15^ Qualitative studies from non-UK countries have also highlighted knowledge gaps (both actual and perceived) among GPs, including lack of risk-factor awareness on how to identify patients in need of assessment for the condition, familiarity with guidelines and policy, and pharmacological management.^16–22^

Although clinicians reported confidence when communicating risk to patients, nurses continued to communicate risks using percentages, which may not be understandable for all patients,^23^ suggesting training in risk communication is needed.^24,25^ Furthermore, fracture risk was not always well understood or recalled by patients, reflecting previous work in this area.^26,27^ Limited recall of fracture risk discussions in patient interviews in this study may be attributable to multiple factors. First, some patients during their interview reflected that the time elapsed since their INCLUDE review hindered their recall. Second, the INCLUDE review covered cardiovascular disease risk, anxiety and depression, and fracture risk, meaning that recalling specific parts of the review may be challenging for patients.^10^ In addition to fracture risk, explanations also need to include understandable descriptions of what osteoporosis is and its consequences to inform patient decision making.^28^ Patients in this study did not report an awareness of the condition and its consequences and reported information needs, which may have influenced their lack of engagement with investigations for osteoporosis. Alternatively, investigations for osteoporosis may not have been welcomed by patients owing to competing healthcare priorities, especially for those with multimorbidity, as identified in previous literature.^29^

Both GPs and nurses were uncertain as to whether it was the role of primary or secondary care to establish osteoporosis risk in patients with IRCs, representing an important management barrier. In other qualitative studies, patients have reported lack of effective communication between primary and secondary care providers.^30,31^ An additional barrier identified in the present study's findings related to the lack of integration of FRAX into electronic GP systems. Nurses expressed frustrations when forgetting necessary patient information (for example, height) when moving from the GP system to the external FRAX website. This finding is supported in previous GP interviews where a desire for FRAX to be integrated into electronic patient records was reported.^22^ Finally, when delivering multimorbidity care reviews focusing on numerous distinct comorbidities, further research is essential to understand competing demands and how to overcome barriers such as potential conflict in clinician and nurse priorities.^32^

### Strengths and limitations

The study has several strengths and weakness. Strengths include the incorporation of multiple methods with both recorded consultations and interviews to explore dissonance and concordance between patient and clinician perspectives. The TDF provided a comprehensive theoretical lens to identify barriers to and facilitators of implementing nurse-delivered fracture risk assessments in primary care, with the deductive framework approach supporting multidisciplinary involvement when interpreting the findings.^33^ All TDF domains, with the exception of behavioural regulation, were used in the deductive analysis. In the study, two of the four nurse interviews were completed before the nurse had conducted an INCLUDE review in practice, with GP participants not completing the reviews, limiting the extent to which behavioural regulation strategies would be discussed during the interviews.^34^ The sample of patient participants included a wide range of ages and different IRCs.

Limitations include that the sole focus of this pilot primary care review was not osteoporosis care, the topic guide was not explicitly developed for exploration of the fracture risk assessment component, and the sample of clinicians was small. The delay between the INCLUDE review and the patient participant interviews may have, as previously outlined, impeded recall and richness of the data collected. These factors mean that data saturation in the clinician interviews was unlikely achieved. However, to the authors’ knowledge, this is the first study of its kind to examine the feasibility of fracture risk assessments in primary care, providing an indication of some of the important barriers. The study was able to demonstrate that a FRAX assessment, in the context of a primary care multimorbidity review, was generally well received by patients and clinicians.

### Implications for research and practice

In this study of the barriers to and facilitators of operationalising fracture risk assessments in primary care, it was found that patients and nurses were positive and clear about the goals of the review and potential benefits. Important barriers related to clinical decision making, risk communication, professional roles, and integration of FRAX with existing IT systems. The experiences of the nurses in this study suggest that with appropriate training, practice nurses are likely to be confident and able to play a key role in conducting fracture risk assessments, but further implementation studies are needed to address the barriers identified.

## Data

The datasets used and/or analysed during the current study are available from Clare Jinks on reasonable request and in line with standard operating procedures on data sharing at Keele University.
